# Enhancing Skin Cancer Immunotheranostics and Precision Medicine through Functionalized Nanomodulators and Nanosensors: Recent Development and Prospects

**DOI:** 10.3390/ijms24043493

**Published:** 2023-02-09

**Authors:** Aisha Farhana

**Affiliations:** Department of Clinical Laboratory Sciences, College of Applied Medical Sciences, Jouf University, Aljouf 72388, Saudi Arabia; afarhana@ju.edu.sa

**Keywords:** skin cancer, immunotherapy, immunomodulation, nanomaterials, novel nanocarriers, melanoma nanoimmunotherpy, precision medicine, theranostics

## Abstract

Skin cancers, especially melanomas, present a formidable diagnostic and therapeutic challenge to the scientific community. Currently, the incidence of melanomas shows a high increase worldwide. Traditional therapeutics are limited to stalling or reversing malignant proliferation, increased metastasis, or rapid recurrence. Nonetheless, the advent of immunotherapy has led to a paradigm shift in treating skin cancers. Many state-of-art immunotherapeutic techniques, namely, active vaccination, chimeric antigen receptors, adoptive T-cell transfer, and immune checkpoint blockers, have achieved a considerable increase in survival rates. Despite its promising outcomes, current immunotherapy is still limited in its efficacy. Newer modalities are now being explored, and significant progress is made by integrating cancer immunotherapy with modular nanotechnology platforms to enhance its therapeutic efficacy and diagnostics. Research on targeting skin cancers with nanomaterial-based techniques has been much more recent than other cancers. Current investigations using nanomaterial-mediated targeting of nonmelanoma and melanoma cancers are directed at augmenting drug delivery and immunomodulation of skin cancers to induce a robust anticancer response and minimize toxic effects. Many novel nanomaterial formulations are being discovered, and clinical trials are underway to explore their efficacy in targeting skin cancers through functionalization or drug encapsulation. The focus of this review rivets on theranostic nanomaterials that can modulate immune mechanisms toward protective, therapeutic, or diagnostic approaches for skin cancers. The recent breakthroughs in nanomaterial-based immunotherapeutic modulation of skin cancer types and diagnostic potentials in personalized immunotherapies are discussed.

## 1. Introduction

The global burden of skin cancer has increased tremendously over the past decades, reaching an epidemic proportion in many regions of the western world [1,2]. It has profoundly impacted public health, impeding socio-economic development in several countries. Skin cancer is primarily presented in keratinocyte carcinoma (nonmelanoma) and malignant melanoma. Keratinocyte carcinoma is further classified as basal cell carcinoma (BCC) or cutaneous squamous cell carcinoma (CSCC). BCC, accounting for 80% of skin cancers, is the least aggressive of all skin cancers and is usually not fatal. CSCCs are limited to the upper and middle layers of the skin and account for 20% of the cases. Though not a life-threatening condition, it can turn aggressive and spread to other parts of the skin if not treated at an early stage. Squamous and basal cell carcinomas are primarily associated with excessive sun or ultraviolet (UV) light exposure. Contrarily, melanomas represent a more heterogeneous and complex cancer type, spanning both benign and malignant cancerous transformations. As a result of its ability to infiltrate various cell types, melanomas are associated with diverse clinical outcomes representing the major cause of mortality due to skin cancers. Melanoma skin cancer (MSC) is developed by uncontrolled proliferation and invasive spreading of abnormal melanocytes. Melanomas are predominantly pigmented, but a small percentage of cutaneous melanomas are amelanotic [3,4], which poses difficulty in its early diagnosis, leading to a delayed therapeutic intervention [5,6]. Melanomas represent the fastest-increasing cancer globally, with an annual increase of 3–8% in the incidence among the European population [1]. Key etiological factors for melanoma include UV and ionizing radiations and chemical carcinogens.

Genetic predisposition with familial germline CDKN2A mutations or sporadic mutations in MAPK pathways is observed in many clinical cases of melanoma [7,8]. BRAF and nRAS mutations are associated with cutaneous melanomas, whereas GNAQ and GNA11 mutations are more common in uveal melanomas [9]. The KIT mutations are predominantly associated with mucosal and acral melanomas [10]. Immunologically distinct expression of programmed cell death protein 1 ligand 1 (PDL1) and programmed cell death protein 1 ligand 2 (PDL2) is observed in various melanoma cells [11]. Immune checkpoints modulation and alteration in the PI3K-AKT-PTEN pathway play an important role in its development and progression [12]. Even with the progress of therapeutic intervention methodologies, melanomas remain the most aggressive skin cancer, with an index of 15–20% five-year survival rate (Cancer statistics, 2019). Presently newer prospects have been explored by the research community to overcome the bottleneck encountered in the theranostics and imaging of skin cancer through the use of functionalized nanomaterials such as liposomes, carbon-based, metal-based, dendrimers, cubosomes, lipid-based, polymer-based, micelles, virus-based, exosomes, and cell membrane-coated nanomaterials [13].

## 2. Immune Mechanisms in Skin Cancers

Immune cells and immune organs protect the overall integrity of the human system through a sophisticated interplay between maintaining tolerance for self-antigens and mounting a robust immune response to eliminate foreign antigens. The two axes of immunity, innate and adaptive immune mechanisms, consolidate the final effect of an immune response. Macrophages, dendritic cells (DC), and natural killer cells (NK) maintain the first line of defense against non-self-antigens, manifested as inflammation. The innate immune response is followed by an intricate network of specific adaptive immune mechanisms levitating the elimination of non-self.

The cancer microenvironment is a non-self component of the body enriched in innate and adaptive immune cells, specifically tumor-infiltrating lymphocytes (TIL) and tumor-associated macrophages (TAM). The cellular composition of the tumor microenvironment (TME) significantly impacts tumor initiation and progression, exerting either an anti-tumor or pro-tumor effect [14]. In all types of skin cancers, infiltration of TAM and regulatory T-cells (Tregs) is observed [15]. Both cell types distinctly induce immunosuppression, thereby promoting tumorigenesis. In nonmelanoma cancers, the tumor microenvironment is specifically enriched in genetically diverse fibroblasts that increase the heterogeneity of tumor-associated antigenic repertoire [16]. Melanomas are infiltrated by macrophage migration inhibitory factors, T-regs, myeloid-derived suppressor cells (MDSCs), and natural killer (NK) cells [17,18]. Nonmelanoma cancers exhibit a strong link with immunosuppressive human microenvironments, which sometimes show regression upon improvement in immune surveillance [19].

**Figure 1:**
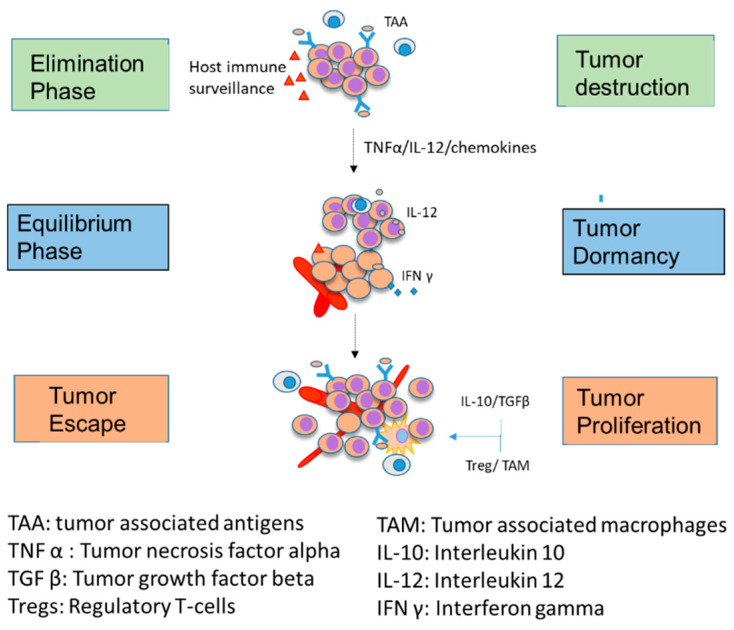
Cancer Immune editing: Cancer cells develop and proliferate through a process of immune editing, a process based on three Es (E^3^), namely, elimination, equilibrium, and escape. The elimination phase is marked by the effective control of host immune surveillance and tumor removal. However, with a breach in immunosurveillance or ineffective host response, the tumor can undergo a dormant phase. Tumor dormancy is reversible and can become active in the presence of pro-tumor factors, leading to tumor proliferation and growth.

Cancer growth and proliferation are maintained by a dynamic process of three phases (elimination, equilibrium, and escape (E^3^), representing the immune editing mechanism) [20]. The response of the immune system to tumor growth is represented by immunoediting, a dynamic process consisting of three distinct phases: elimination, equilibrium, and escape of the cancer cells (Figure 1). Effective host innate and adaptive immune surveillance can trace and destroy tumor cells during the elimination phase. Some immune-resistant variants that emerge in the tumor microenvironment can partially evade the innate and adaptive response and progress to equilibrium. The immune effectors cannot eliminate these variants but render them dormant. The stand-off between the host immunity and tumor cells can sometimes lead to evasion of the immune control, enabling tumor cell variants to proliferate unrestricted, establishing a full-fledged tumor or proliferating cancer (Figure 1).

Dendritic cells (DC) are the major players in counteracting immunosuppressive melanoma and nonmelanoma cancers. Through a functional intersection of cytotoxic T-cell and immune checkpoint receptors, DC can skew the immunosuppressive tumor microenvironment involving endothelial cells, TAM, T-regs, MDSCs, and NK cells toward immune infiltration and immune responsiveness [14]. This disrupts the tolerogenic nature of the tumor microenvironment, potentiating the elimination of the tumor. DC also interacts with Toll-like receptors, ligand-inducing type-1 interferon (IFN), and several proinflammatory cytokines and chemokines. TLR ligation promotes DC maturation, enhancing their antigen presentation potential. Effective antigen presentation of immature T-cells is the hallmark for optimal activation of adaptive immunity and hence tumor regression.

In addition, the DC interaction with TLR ligands induces the production of type-I IFNs and other proinflammatory cytokines within the TME [21]. This interplay also promotes DC maturation and enhances antigen presentation ability to naïve T-cells, thus effectively activating adaptive immunity [20]. A notable feature of effective immune filtration to curtail tumor proliferation is the optimal density, composition, and distribution of cytotoxic T-cells in the stroma, facilitating tumor regression and enhancing the therapeutic efficacy of the drugs and vaccines [22].

## 3. Classical Diagnostic and Treatment Modalities for Skin Cancers

Accurate skin cancer diagnosis remains critical to deciding a successful line of therapy. Classical diagnostic procedures include invasive and noninvasive methods [23]. Both methods encounter complexities and challenges of under or over-diagnosis, and identifying the right cancer type requires considering alternative modalities. Diagnostic approaches for nonmelanoma cancers (BCC and CSCC) usually require a skin biopsy and histopathologic examination to observe the tissue for any cancerous growth [24].

### 3.1. Skin Biopsy

Skin biopsy is also a therapeutic approach for nonmelanoma cancers if the entire tissue needs to be removed. There are many types of skin biopsies, such as shave (tangential) biopsy, punch biopsy, and excisional and incisional biopsies [25]. Shave biopsy is suitable for monitoring the top layers of the skin, punch biopsy extracts deeper skin layers, excisional biopsy removes the entire area of the tumor and, incisional biopsy extracts a small portion of the tumor. Since BCC and CSCC commonly do not spread beyond the skin tissue, alternative or additional diagnoses are usually not considered; however, if a spread is suspected, a lymph node biopsy is carried out to confirm through fine needle aspiration (FNA). Small fragments of lymph nodes are removed and microscopically analyzed.

### 3.2. Noninvasive Techniques

Many noninvasive techniques are now used to detect skin cancers with high accuracy. These techniques include dermoscopy, reflectance confocal microscopy, optical coherence microscopy photography, spectroscopy, thermography, multispectral imaging, electrical bio-impedance, and computer-aided techniques [26,27].

Dermoscopy: The commonly used dermoscopic techniques enable the identification of specific features of skin lesions. It supplements biopsy techniques by providing a complete assessment of the skin lesion to identify the most suitable biopsy sampling site. Melanoma and nonmelanomas are discreetly identified based on specific dermoscopic patterns in the lesions [28,29,30,31]. Though an effective noninvasive diagnostic tool, dermoscopy can only be applied to the upper dermis [27].

Reflectance confocal microscopy: Reflectance confocal microscopy (RCM) is a comparatively new technique for assessing and diagnosing skin cancers. The method harnesses the high reflective qualities of keratin and melanin in the skin tissue. Through the infrared light source, RCM facilitates the visualization of deeper layers of the skin and hence is considered an optical biopsy. Nonetheless, accurate diagnosis through RCM necessitates technical proficiency [32,33].

Optical coherence tomography: Optical coherence tomography (OCT) is another technique to assess skin tumors. Low-power infrared laser light captures real-time images up to 2 mm underneath the skin surface [34]. The technique requires no pretreatment. Thus far, the technique has been used to diagnose basal cell carcinomas; however, the diagnostic accuracy of malignant melanomas has been minimal [35].

Photodynamic Visualization and Therapy: Photodynamic visualization (PDV) is a noninvasive fluorescent laser-based mechanism for monitoring skin cancers. A photosensitizer is targeted at the cancer site and visualized using a fluorescent light source. Conventional methods use 5-aminolevulinic acid in a gel form applied to the skin, which is converted to colored protoporphyrin IX (Pp-IX) in the cells [36]. The photoreactive and physical features of Pp-IX facilitate its clinical use in diagnosis and therapy for skin cancers. Other molecules including cysteine cathepsin proteases and adjuvants such as Vit D are also used as selective fluorescence probes and adjuvants [37,38]. PDV is useful in assessing nonmelanomas; however, identifying melanomas through PDV has provided variable results. Currently, many photosensitizers are developed as nanoparticle formulations or as chemicals. The development of new photosensitizers, porphylipoprotein and talaporphyrin sodium, metal complex base, aggregation-induced emission, and doped carbon dot-based photosensitizers have received considerable attention in cancer treatment [39,40,41,42]. The use of nanoparticle-based PDV has enhanced the rate of detection of nonmelanoma and melanoma skin cancers with promising therapeutic potential [43,44]. Photosensitizers are also employed as a therapeutic mechanism against skin cancers. Photodynamic therapy uses specific photosensitizers that are first targeted to the tumor tissue. Subsequently, fluorescence activation of photosensitizers converts them into cytotoxic reactive oxygen species (ROS) to induce targeted cancer cell death. Technical caveats, such as lack of effective targeting and availability of enhanced and safe photosensitizers, have impeded the use of PDT in cancer cell therapeutics. However, the recent incorporation of nanomaterials for skin theranostics has successfully overcome many technical and therapeutic bottlenecks [45].

Immunodiagnostics and Immunotherapy: Immunodiagnosis of skin cancers has been tested since the 1970s with encouraging results in staging and clinical management of the disease [46,47]. Tumor-specific antigens are identified within the tumor microenvironment through specific monoclonal antibodies. Nonetheless, the expression of antigens within the tumor site and across different patients has shown variation in antigen expression [47]. Hence, proper diagnosis using antibodies requires screening for more than one tumor-specific antigen [48]. Furthermore, diagnostic modalities such as radioactivity have limited the use of immunodiagnosis as a common diagnostic method for skin cancer. Currently, combinational approaches are being studied to maximize the diagnostic potential of immunological diagnostic techniques. Immunobiological loaded microneedle devices are used as diagnostic and therapeutic modalities [49,50]. These microneedles deliver the loaded drug/antibodies in deeper layers of the skin, which are released near tumor cells as a means of tumor detection [50]. Microneedle devices can also synergize with other therapeutic and diagnostic interventions, such as photothermal or photodynamic visualization, for enhanced results [51].

The immunotherapeutic treatment method depends upon the modulation of the host immune mechanism to induce regulatory, stimulatory, or suppressive responses toward the tumor. The host anti-tumor responses are activated through innate immune mechanisms that increase the number of immune effectors or subdue the suppressive tumor microenvironment [52]. Strategies to modulate immune response include immunomodulatory molecules, monoclonal antibodies, therapeutic vaccines, small immunomodulatory molecules, etc. [53]. These molecules facilitate antigen uptake, optimal processing, and presentation to naive T-cells, activating and expanding the repertoire to naïve T-cells. Finally, an intense effector response is launched against cancer cells. Though an effective strategy observed in patients, immune activation can sometimes reach abnormally high levels, risking adverse immune effects. Furthermore, poor therapeutic targeting and insufficient understanding of the immunological skew within the TME have limited immunotherapy for many cancers. Additionally, advanced delivery systems for site-specific targeting of immunotherapeutic agents are needed [52]. Skin cancer immunotherapy as a treatment modality has been much more recent compared to other cancers. It has evolved with the improvement in understanding the immunologic features of nonmelanoma and melanoma cancers. This treatment method for skin cancers has been a paradigm for cancer immunotherapeutics, especially due to their T-cell immunogenicity. Immunotherapy for skin cancers has shown higher success rates than other cancers. Therapy using anti-PD-1 (anti-programmed-death 1) for melanomas, BCC, CSCC, and Kaposi’s sarcoma has shown a 40% response rate [54,55]. Cytokine therapy using Type-1 interferon and interleukin has been tested, but high toxicity and low response rates limited its use in mainstream therapeutics [56,57,58]. 

## 4. Nanomaterials in Diagnosis and Immunotherapy of Skin Cancers

Nanomaterials are versatile small molecules with at least one dimension ranging in size from 100 nm. Nanoparticles harbor an inherent capacity to interact with the effectors of immune response and can modify their functions to cause immunostimulation or immunosuppression [59,60]. Depending upon the type and progress of the disease, the response can be either harmful or beneficial. The structure of nanomaterials, such as size, composition, surface chemistry, and molecular interactions with the target cell, propels useful or toxic immunomodulation [61,62]. As a consequence of interaction with cellular components, unanticipated reactions such as hypersensitivity or inflammation may also result [63]. Ensuring an effective and safe clinical result necessities a thorough understanding of the structure-function chemistry of nanoparticles at a cellular interface. Nanoparticles can incorporate many functional components, demonstrate spatiotemporal regulation [64,65,66], and facilitate the topical delivery of drugs, vaccines, and therapeutic and diagnostic material. With enhanced theranostic potential and minimal toxicity, topical delivery has been sought after, especially in skin diseases and cancers. Currently, nanosystems are extensively investigated for their potential to improve or modulate immune responses against cancer, imparting cancer protection or preventing recurrence. Though only a few nanomodulators have been tested on different skin cancers, a sudden thrust is observed in nanomaterial-based theranostic studies against skin cancers.

Recently, computational approaches, such as Box Behnken and Central Composite Design, have been integrated into simulating the molecular dynamics, assessing resilience and interaction of nanoparticles with the cellular microenvironment [67,68,69]. This has expanded the repertoire of nanomaterials and nanosystems to be tested for cancers. This design of experimental methodology enables in silico assessment of area-to-volume ratio, optimal drug loading and controlled release, tensile strength, in vivo safety and stability, etc., of the nanomaterial. Computational designs have introduced a breakthrough in developing nanoparticle-based next-generation interventions such as drugs and vaccine targeting, immunomodulation, and drug discovery, reducing the bench-to-bedside gap [70,71].

**Figure 2:**
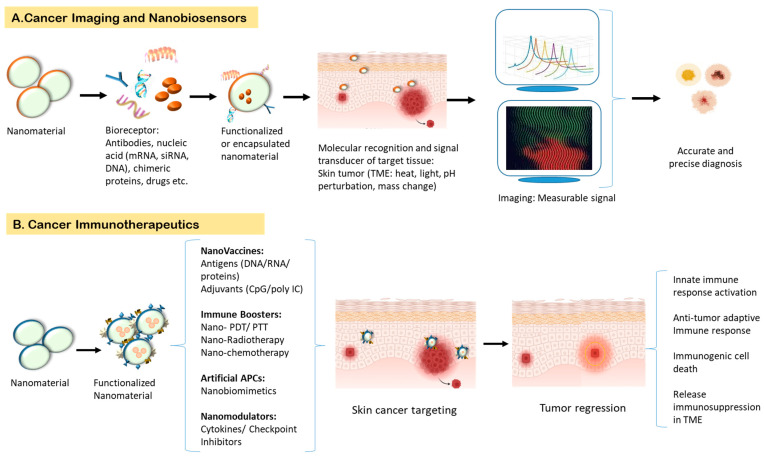
Mechanism of cancer diagnostics and therapeutics using nanosystems. (**A**) Nanomolecules can be functionalized with specific modulators (such as antibodies, nucleic acids, proteins, etc.) and targeted to specific cancer sites. Based on their ability to sense perturbations in pH, temperature and concentration changes, etc., signals can be generated and measured to precisely and accurately diagnose the type and stage of cancers (**B**) Nanosystems can be targeted to cancer microenvironment as a therapeutic modality to induce tumor regression.

Nanoparticles demonstrate an exclusively enhanced permeability and retention (EPR) effect in addition to substantially reduced cytotoxicity and property of functionalization. Nanosystem-based skin cancer therapeutic approaches span carcinoma targeting nanoparticles, specific cancer cells targeting functionalized nanomaterials, and active targeting through receptor-mediated delivery. The repertoire of nanoparticles for skin cancer targeting is extensive, encompassing nanoshells, liposomes, ethosomes, nanostructured lipid carriers, polymeric nanoparticles, nanospheres, solid lipid nanoparticles, fullerenes, dendrimers, quantum dots, carbon nanotubes, etc. (Figure 2).

Skin cancer therapy has harnessed the potential of nanosystems with good efficacy to overcome several bottlenecks of conventional therapy and underdiagnosis in the research setting. Many types of nanomodalities have been tested for skin cancers, especially melanomas. Nanomaterials used for skin cancer therapeutic span a wide range, including liposomes, carbon nanotubes, dendrimers, metallic formulations, and protein-based nanoformulations. Currently, graphene and graphene oxide nanocomposites have demonstrated efficacy as optical and electrochemical biosensors for the detection of early stage cancers. The ultraslim dimensions and excellent electrical and thermal conductivity, and mechanical tensility make graphene nanoparticles a unique biomolecules and cellular sensing tool for early cancer detection [72,73]. Recently, local applications of drug or immune-based nanoformulations for skin cancer therapeutics have been explored with considerable success. Topical delivery is a promising alternative to generalized therapy with reduced systemic toxicity, irritation risk, and optimal permeation [74]. Furthermore, drug resistance in cancers can pose therapeutic ineffectiveness, and hence various drugs and dosages need to be tested in nanoparticles. It is evident that currently, nanotheranostics in skin cancers is at its initial stage demanding animal and clinical evaluations before translating into clinical use.

## 5. Nanomodulation of Mitochondrial Function as Immunotherapy against Skin Cancers

Similar to other cancers, the initiation and progression of skin cancer are also modulated by mitochondria-driven energy metabolism [75]. Mitochondrial regulation of TME and the development of metabolic heterogeneity substantiates cancer cell proliferation and immune evasion [76]. Cancer cells circumvent cellular apoptosis and maintain a high biosynthetic and bioenergetics potential, carefully regulated by mitochondria. Owing to the pivotal role of mitochondria in metabolism, oxidative and nitrosative stress management, bioenergetics, and modulation of apoptosis targeted nanotherapies to reverse program TME, and immune evasion can potentiate effective cancer management and therapy. Targeting mitochondrial functions through drug and biomolecule-encapsulated nanomaterials has the potential to initiate a ripple effect in the tumor microenvironments, promoting a therapeutic response [77,78,79].

In mice models of melanomas, mitochondrial complex-I targeted therapy has shown promising effects in abrogating cancer progression through a concerted effect on oxygen consumption, cell signaling, and cell cycle [80]. It is demonstrated that cancer cells develop nanotubes to physically extract mitochondria from immune cells. This unique hijack machinery within the TME metabolically empowers the cancer cells in addition to depleting immune cells [80]. Hence, mitochondria-mediated crosstalk between the immune and metabolic components of cancer presents mitochondrial targeting by nanomaterials as one of the major cancer therapeutic strategies. Currently, CAR (chimeric antigen receptors) T-cell therapy has revolutionized the treatment of various cancers. Many clinical trials with CAR T-cell therapy for various cancers are under clinical trials (NCT04348643, NCT04348643, NCT01454596, NCT04877613) [81]. However, adoptively transferred cells do not consistently demonstrate effective persistence in patients. Nanomaterial-mediated targeting and reprogramming of mitochondrial pathways central to the function and survival of T-cells may facilitate effective results in patients.

## 6. Nanomaterials Enriching the Repertoire of Immunotherapeutic Modules against Skin Cancer

Nanomaterial-based nanosystem applications have been incorporated into many therapeutic modules for almost all types of cancers. Nanosystems have been effectively used in drug delivery, tumor targeting, immunomodulation, cellular imaging, and image-directed tumor ablation (Figure 2). Nanoparticles can be customized according to the therapeutic need (Table 1). They can circumvent biological barriers and deliver drugs and other therapeutic agents to the targeted tumor microenvironments, thereby escalating the treatment efficiency and reducing side effects. Nanoparticles can easily penetrate the skin and deeper tissues and be propelled to specific tumor sites for targeted delivery of encapsulated drugs or other biomolecules [82]. Recently, extensive research efforts have been channelized to develop effective nanoparticles with the potential to functionalize them according to the theranostic need. Recent research has diversified from the traditional use of nanoparticles in drug delivery and biodistribution to channelize and skew immunological responses beneficially. Though some nanoformulations for cancer therapy have reached clinical use, most nanotherapeutics are in the developmental phase [83] (Table 1).

### 6.1. Liposomes

Attributable to their ability to encapsulate drugs and antigens with different physicochemical properties, functionalized liposomes have been used in cancer therapy as targeted delivery vehicles [84]. The liposomal surface can be modified by pH and fusogenic material to achieve targeted antigen delivery, leading to the cross-presentation of exogenous antigen through the cytoplasmic pathway [85]. Several bottlenecks that are encountered in cancer immunotherapeutic approaches can be resolved through liposome-based systems. Liposomes can improve antigen delivery by directing it to surface receptors on APC/T-cells by selective release of antigens for an effective cross-presentation, improving the vaccine potential. Toll-like receptors, bioactive polysaccharides, and lipids as adjuvants can enhance the immune-activating potential of the liposomes. Adjuvants that counter immunosuppressive tumor microenvironments can enhance the anti-tumor effect of the antigen-loaded liposomes. Simultaneous delivery of liposome-encapsulated drugs in combination with radiotherapy, chemotherapy, and radiotherapy can lead to better results [86]. Currently, metal complex-based liposomes have been developed to maximize the therapeutic potential and reduce side effects [85]. These complexes are under clinical trials in many immunological applications for enhanced cellular targeting and drug delivery. Skin cancer immunotherapy can benefit from developing a purposeful design of metal-complexed liposomes targeted to immunosuppressive melanoma microenvironments [85].

### 6.2. Metallic Nanoparticles

Metallic nanosystems used in cancer theranostics include aluminum oxide, iron oxide, gold, silver, titanium dioxide, and zinc oxide nanomaterials for immunomodulation [18,87,88,89,90,91,92]. A diverse array of immunotherapeutic applications, such as the delivery of immunomodulators, induction of tumor-dependent antigen release, targeted vaccine therapy, improved antigen presentation, ROS generation, perturbing tumor microenvironments, etc., have successfully utilized metallic nanoparticles [93,94]. Silver nanoparticles functionalized with bovine serum albumin coating (BSA) demonstrated multimodal therapeutic potential. These particles visibly show cytocidal activity in melanoma cultures and promising inhibiting effects on angiogenesis in vitro. These BSA-ligated silver nanoparticles also exhibit a marked light-to-heat conversion ability and hence could be used in photothermal therapies for skin cancers [92]. Gold nanoparticles (GNP) are also prominent in cancer targeting and immunotherapy [95,96]. As excellent biocompatible materials, GNPs are efficient antigen and adjuvant delivery vehicles for inducing a cytotoxic T-cell response, can artificially present antigens, and can be functionalized to provide co-stimulation. GNP can bind with human DC to help activate cytokine production [97]. GNP demonstrates a high plasmonic effect, reduced cytotoxicity, and ability of diverse functionalization [98,99]. Cancer immunotherapy has used GNPs in combination with TNF-α, TGF-β, the PD-1 inhibitor, TLR-7 agonist, specialized antibodies, and other tumor cell death factors or/and immunostimulants. Iron oxide (ferrites) based metallic nanoparticles can be molded into magnetite nanosystems. These nanoparticles comprise a magnetic inner shell coated with specific immunomodulators. Magnetic nanoparticles have proved effective in magnetic hyperthermia-mediated cancer immunotherapy [100,101]. Fe_3_O_4-_SiO_2_ cancer-specific antigens functionalized magnetic nanosystem encapsulated in the cell membrane demonstrated a pronounced NK cell-based immunotherapy [102]. Drug-functionalized Ferric oxide (FeO) nanoconjugates have proven effective in modulating cancer cell apoptosis, metabolic reprogramming, DNA toxicity, etc. [103,104,105].

### 6.3. Carbon Nanotubes

Carbon nanotubes (CN) are carbon-based cylindrical single-walled or multi-walled carbon tubes with exceptional thermal, optical, and electronic conductivity, which enables diverse functionalization and loading potential. These nanotubes can achieve high targetability within the tumor cells and in the extracellular tumor microenvironment [106]. Pertinent to their extraordinary hydrophobic feature transport of drugs, biomolecules and sensor compounds can be easily targeted in a site-specific manner. Furthermore, the ease of functionalization with various biomolecules and biomimetics for sensing, imaging, and therapeutics makes CN one of the major players in cancer theranostics spanning diverse cancers [107].

CNs can target tumor antigens’ (ovalbumin, cytosine-phosphate-guanine oligodeoxynucleotide, etc.) presentation of APCs [108]. Doxorubicin-conjugated CN can be targeted at melanoma sites to induce cell death [109]. Polypyrrole-coated carbon nanotube composite is recently synthesized as an effective sonosenitizer, wherein low-intensity UV irradiation can enhance its treatment potential [110]. Carbon nanotubes recreate properties such as cell transport and targeting. CN, especially single-walled nanotubes, can be used in conjunction with photothermal therapy, potentiating an enhanced targeted treatment [111].

### 6.4. Polymeric Nanoparticles

Polymeric nanoparticles (PN) are biodegradable polymers used in cancer theranostics with a unique capacity for surface functionalization, stability and malleability of size and morphology, and efficient therapeutic payload potential. Various biodegradable polymers are used in the synthesis of polymeric nanoparticles, such as poly(lactide) (PLA), poly(lactide-co-glycolide) (PLGA) copolymers, poly (ɛ-caprolactone) (PCL), and poly(amino acids) and some natural polymers such as alginate, gelatin, albumin, etc. PN is generally used as a pH-sensitive modality to administer drugs and immunotherapeutics [112]. Immunodiagnostic approaches with PN have significantly enhanced computed tomography imaging and targeted chemotherapy of melanomas. Bioadhesive PN-based camptothecin targeting can be achieved by PLA attached to hyperbranched polyglycerol (HPG). The PLA-HPG nanosystem showed selective binding to the SCC tumor cells and matrix. This binding enhanced targeted intratumor delivery and therapeutic efficiency of the drug [113]. Targeted PN functionalized with oncolytic peptide LTX-315 substantiated with CpG adjuvant and PD-1 antibody system resulted in long-term immunotherapeutic effects in mouse models [114].

(R848)-loaded PN was demonstrated to target the mitochondria of melanoma cells effectively. Combined with near-infrared photothermal therapy and immune checkpoint blockers, the (R848)-loaded PN facilitated tumor immunosuppression, subsequently achieving anti-tumor effects [75]. PGLA encapsulating TLR7 and TLR8 agonists increased cytokine secretion through its effect on APC. Through enhanced co-stimulation, the nanoparticles led to the expansion of CD8^+^ T-cells and substantiated CTL response, resulting in significant therapeutic efficacy in melanomas and other cancers [115]. Though exceptional delivery vehicles, PN also exhibits high cytotoxicity and poor stability in vivo, requiring further technical reprogramming to be effectively translated to clinical settings.

### 6.5. Dendrimers

Dendrimers are compact, biocompatible, and highly branched artificial molecules with modifiable functionality. Dendrimer-based nanosystems can maneuver different biological barriers in the bloodstream with the least effect on their efficacy. Effectively functionalized dendrimers and dendrimer hybrids have been investigated as promising immunotherapeutics. They can combine numerous functional groups within their compact molecular dimension. Many types of dendrimers, such as poly(phosphorhydrazone) (PPH) dendrimers (ABP)-capped phosphorous dendrimers, aza-bisphosphonate, etc., are being tested. Glycodendrimers have enormous potential to activate Th1 response and NK cell activity [116]. Due to their affinity for lectin-like receptors, glycodendrimers can enhance DC cross-presentation of antigens, inhibit chemokines and cytokines production, and enhance anti-tumor immunity within the tumor milieu [117]. Lactose-terminated dendrimers can produce a robust immunostimulatory effect by activation of signaling response that functions through NFAT, NFκB, and AP-1 pathways. ABP-capped dendrimers potentiate an anti-inflammatory effect through the induction of IL-4, IL-10, and IL-13 cytokines and decreasing inflammatory surface CD64 and CD13 [118,119].

Furthermore, through its interaction with monocytes, ABP-capped dendrimers subdue CD4^+^ T-cell proliferation and NK cell activity. Carbosilane dendrimers limit M2 macrophage polarization by reducing IL-10 production in M2 macrophages. This favors anti-tumor response by switching M2 macrophage phenotype to M1, thereby facilitating an anticancer response [120]. PAMAM G3 dendrimers substituted with R-glycidol and celecoxib/simvastatin demonstrate an efficient, targeted drug delivery potential and can be tested for immunotherapeutics [121].

## 7. Pharmacokinetics and Toxicology of Nanomaterial Repertoire Enabling Cancer Theranostics

The incorporation of various nanoformulations in cancer theranostics has diversified and enhanced the potential of skin cancer immunotherapeutics and personalized medicine. Nonetheless, their inherent small size and high cellular permeability can alter pharmacokinetic effects and toxicity. Researchers have tediously worked to optimize nanoformulations for various medical uses, especially cancers.

Liposomal nanoparticles have a huge repertoire generated by combinations of cholesterol, phospholipids, polyethylene glycol, etc., imparting diverse functionality and use in different TME and cellular targeting. Though the physiochemical properties of liposomes are the least pharmacologically active and well tolerated in many models, the type of targeted environment, dose and time of exposure, and surface functionalization can impart toxicity [122]. Liposomes are demonstrated to interact with healthy cells and proteins in circulation, altering their functions. Animal studies have shown cell-dependent toxicity of the drugs and nucleic acid-loaded cationic liposomes, which were toxic to macrophages and monocytic cell lines but not to T-cells. The toxicity increased with higher zeta potential and some combinations of liposomal constituents [123]. Liposomes can interact with the mononuclear phagocyte system (MPS) [124]. Thus, in addition to the target cells, the therapeutic payload is directed to the phagocytic cells of the lymph nodes, bone marrow, liver, and spleen, impairing their functions. Few studies have also demonstrated that the dose of the therapeutic drug and composition of the liposome can cause aggregation and induction of edema, necrosis, and inflammation at the site of the entry. A meta-analysis study has evaluated that an average of 1% of the nanoparticles reach the targeted tumors [125,126]. Hence, systemic effects remain a challenge that needs to be addressed by various in vitro toxicology assessment methods. Some liposomal drug formulations are also associated with the induction of complement pathways and hypersensitivity reactions in addition to increased blood clearance [127,128]. Overall, the full potential of liposome-based immunotherapeutics can be achieved through careful assessment and understanding of various formulations of liposomes with the cellular and immune machinery.

Metallic nanoparticles such as superparamagnetic iron oxide, which were FDA-approved, and widely used diagnostic nanomaterials, have been pulled off due to their toxic potential. Studies have noted ROS production, inflammation, lactate dehydrogenase leakage, mitochondrial dysfunction, and DNA strand breaks due to some metallic nanoparticles. Metal and metal oxide nanoparticles of Ag, TiO_2_, Ni, and ZnO translocate to the lung and gastrointestinal system and reach systemic circulation further accumulating on MPS and the liver [129]. Metallic nanoparticles also demonstrate a high potential to accumulate in sub-cellular organelles. Lysosomal interaction of metallic nanoparticles leads to ROS production, and the release of reactive ions that may inflict DNA damage, inflammation, and genotoxicity [130]. Polymeric nanoparticles have a wide application in cancer drug delivery and diagnostics. In spite of being the most tunable nanomolecules, it has a broad range of associated toxicities. The adverse effects of polymeric nanosystems are due to the quantum size effects, monomer aggregation linked to ROS generation, cytotoxicity, and DNA damage, in addition to their toxic degradation mechanism [131]. Cancer theranostics have harnessed CN as a modular platform due to their adaptable physicochemical and mechanical properties. Nevertheless, these advanced properties of CN also are a cause for concern. CNs have been tested as modular drug trafficking modalities for cancers in animal models. Some studies record that CN can induce immunotoxicity, neurotoxicity, and toxic effects on the lung, liver, cardiovascular system, etc [132]. Small molecular weight CN can induce inflammation with slow recovery in addition to the generation of ROS and induction of inflammatory responses [132]. Developmental toxicity was also observed in mice with pronounced teratogenic effects, fetal malformation, and miscarriages. In mice, CN demonstrated the development of atherosclerotic plaque, arrhythmia, and vasomotor dysfunction. However, the toxic impact of CN has thus far been associated with its size and high surface area [133]. Studies to modulate the synthesis, functionalization, and encapsulated drug concentrations and assessment of cytotoxicity in animal models are crucial.

Toxicology studies on generation 4, 5, and, 6 PAMAM dendrimers indicate detrimental effects on mammalian cells in a dose-dependent manner [134]. The toxicity assessment in human intestinal, endothelial, and skin cells demonstrated a correlation with the number of amine groups on the dendrimer surface [135]. Studies have indicated that surface modification that modifies the surface to a neutral or anionic state can reduce the toxicity index of PAMAM dendrimers. Studies have shown the toxic effects of dendrimer spanning aggregation of many blood proteins and ROS generation leading to DNA damage [136]. Generation-5 PAMAM dendrimer, when administered through the intranasal route, can lead to acute lung failure [137]. PAMAM dendrimers with amine termini have higher toxicity as compared to PAMAM dendrimers with carboxylic acid termini. Studies on surface modification, appropriate dosage, and therapeutic methodology assessment can enhance the biocompatibility of PAMAM dendrimer.

## 8. Nanoimmunotherapeutics and Precision Medicine in Skin Cancer: Research and Clinical Trials

Nanosystem-based immunotherapeutics for skin cancer manifests either through a potent anti-tumor immune response or channelizing immune defense mechanism to disrupt the immunosuppressive nature of the tumor. Several nanoparticle-based immunotherapeutic modalities have been tested, and some have reached clinical trials. A few effective modalities, such as cancer nanovaccines, immune checkpoint inhibitors, oncolytic virus therapy, and adoptive cell transfer, which have enhanced skin cancer therapy by breaching the therapeutic bottleneck, are discussed.

### 8.1. Nanoparticle-Formulated RNA

RNA-based immunomodulation of cancers is now being explored to enhance the therapeutic efficacy of existing immunotherapy. Many modalities are used, such as regulating cellular function through mRNA, siRNA, or stimulating immune components, specifically TLRs or cytosolic RIG-I [81,138,139]. RNA-based immunomodulation can be achieved to full potency only through nanoparticle encapsulated delivery systems, which facilitate overcoming ineffective target cell delivery, degradation through RNases, cross membrane diffusion difficulties, etc. Many materials have been tested for delivering mRNA including lipids, lipid and protein derivatives, and polymers [140,141,142,143]. Lipid nanoparticle encapsulated mRNA vaccines have shown promising results in diseases as diverse as viral and bacterial infections and cancers. However yet to be established in skin cancers [144,145]. The ability of mRNA to translate to protein directly precluded many bottlenecks associated with conventional therapeutics besides a higher potential to activate immune components with sustained responses. mRNA-based therapeutics also limit the risks associated with radiation and chemotherapy and are among the safest vaccine approach [81]. Usually modeled using mRNA encoding TAM, mRNA vaccines can manipulate immune mechanisms, leading to long-standing responses by activating memory T-cells [95]. Lipid- mRNA formulations have proven efficacious in preclinical testing. These formulations target mRNA to DC and facilitate its endocytosis. The intracellular delivery in mRNA targeting can be achieved using the functionalization of pH-sensitive lipids or polymers. Furthermore, it helps escape mRNA from endosomal degradation [143].

**Figure 3:**
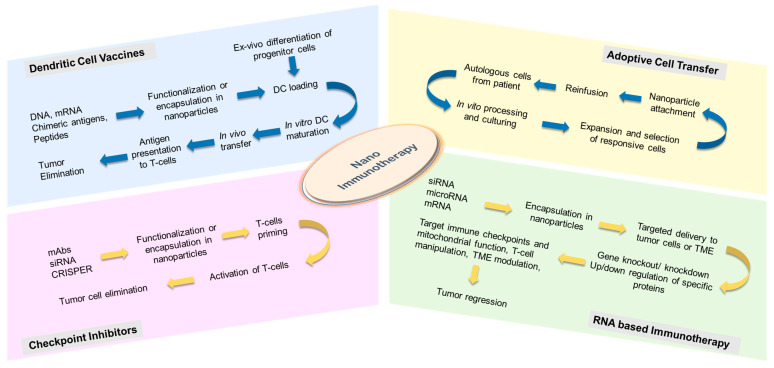
Nanoimmunotherapy interface for cancers: Immunotherapeutics for cancers have used nanomaterials to boost the potential of various immunotherapy techniques such as DC vaccine, adoptive cell transfer, checkpoint inhibition, and RNA-based immunotherapeutics. The basic mechanisms are illustrated.

Considering its ability to circumvent mRNA instability within the cellular milieu, mRNA as skin cancer vaccines hold an enhanced potential as a therapy. Some mRNA vaccines have been tested in patients. These vaccines can be made in vitro without using inactivated pathogens or particulate material as conventional vaccines [146]. This enables customization to incorporate any specific nucleotide sequence. Nanoparticles containing mRNA-based treatment for skin cancers, especially melanomas, may provide better therapeutic avenues with fewer side effects and higher curative potential [147] (Figure 3). Some mRNA nanodrugs for the treatment of melanoma are now in clinical trials [148]. Nanoparticle-assisted delivery system-based RNA therapeutics is expected to offer tremendous potential for treating skin cancers.

### 8.2. Nanoparticle-Enabled Dendritic Cells Vaccines

Dendritic cell (DC) vaccines are currently used for melanoma therapy. Being the most important antigen-presenting cells, DC can help regulate immune activation and overcome tolerance to modulate cancer immune responses. DC can act as adjuvants in initiating immune responses or as effectors to redirect cytotoxic CD8+ T-cells against melanoma. Unlike other skin cancer immunotherapy approaches, the percentage of patients that can benefit from DC therapy is higher, owing to their potential to overcome distinct immunosuppressive or immunotolerant tumor microenvironments [149,150]. To enhance the potential of DC as therapeutic vaccines, modulation of their outer structures with modular nanomaterials has gained interest. DC can be appropriately programmed ex vivo for adoptive vaccination to induce specific in vivo anti-tumor immune responses. DC vaccines require isolating or in vitro culturing precursor cells from peripheral blood and loading them with tumor-associated antigens. These DCs are matured through the application of specific stimulatory molecules [151]. Many technical strategies have been explored to effectively potentiate DC to engender a directed and robust anti-tumor response. Integration of methodologies that can orchestrate efficient tumor antigen cross-presentation, T-cell co-stimulation, effector cell polarization, targeted migration, and avoiding immunodominance of DC are needed to maximize the potential of DC vaccines. Manipulating DC vaccines through the spatiotemporally controllable and modifiable function of nanomaterials has shown promise against skin cancers in both in vitro and in vivo studies [152] (Figure 3). Combination therapy with monocyte-derived DC vaccines can be loaded with preferentially expressed antigens of melanoma or whole apoptotic cell substances, cell-derived mRNA. This nanoformulation can be targeted in a site-specific manner through functionalized nanoparticles [153]. Silencing PD-1 in DC vaccines increases CD8+ priming [154]. Antigenic RNA silencing PD-1/2 through nanoparticle vehicles that translocate siRNA to DC can induce robust CD8+ stimulation [155]. Nanoparticle-based mRNA-transfected DC maintains the phenotypic nature and migration potential of DCs [156]. RNA-modified DC vaccine can also help overcome tumor resistance to therapy generated by auto-inductive loops developed by checkpoint inhibitors and tumor milieu, making skin cancer resistant to many traditional treatments [157]. Neoadjuvant and neoantigens obtained from tumor sites loaded in DC cells as effective vaccines [153]. The use of nanomaterial in DC-based therapy allows for the incorporation of various functional molecules to enhance antigen presentation and overcome several methodological deficiencies [158,159]. Biocompatible nanomaterials also alleviate the toxicity of the treatment modality, besides increasing the therapeutic effect of cancer DC [160]. Nanoencapsulation improves the biophysical-chemical properties of antigenic formulations and imparts quality enhancement, antigen protection, and antigen presentation. Furthermore, DC can be conjugated with functional molecules to mediate crosstalk with APC and TME to complement and reinforce DC-based immunotherpeutics. Nanoparticles associated DC are expected to increase their in vivo circulation span and limit their degradation.

### 8.3. Nanoparticle-Based Immune Checkpoint Inhibitors

Immune checkpoint receptor pathways are immune synapses that directly or indirectly orchestrate cell-to-cell communication. In cancers, immune checkpoint inhibitors (ICI) abate T-lymphocyte responses and skew the deregulated immune system to induce CD8^+^ T-cell mediated cancer killing. FDA-approved monoclonal (mAb) anti-CTLA4 and anti-PD-1 antibodies as ICI have been successful in the management of advanced melanoma. Based on a patient study, anti-CTLA and PD-1 have shown significant tumor regression and long-term cancer management in nearly 50% of patients [161]. In a retrospective cohort (2010–2019) study comprising 16,831 metastatic melanoma patients, the use of ICI in addition to immunotherapy showed a marked improvement in survival [162]. Anti-CTLA4 and anti-PD-1 therapy have demonstrated the therapeutic promise of overall 5-year survival in advanced melanomas, with the curtailment of brain metastases [163]. The addition of an anti-LAG3 antibody to combined anti-CTLA4 and anti-PD-1 therapy demonstrates a progression-free survival, as observed in phase 3 trials. The use of PD-1 inhibitors is established as a standard of care in stage III or IV high-risk melanomas as adjuvant therapy [163]. Nonetheless, the development of autoimmune toxicities associated with immune-related adverse effects is some of the drawbacks of ICI requiring immediate management. Being different from an actual autoimmune disease, these adverse immune events have no specific management available [164]. The intrinsic property of the nanomaterial is harnessed to ameliorate potential injuries and adverse reactions and potentiate long-lasting responses. Nanoparticles can be functionalized to precisely and accurately target the delivery of more than one ICI and can be specifically localized to inhibitor or both stimulatory and inhibitory checkpoints. Nanosystem-based ICI treatment can enhance the bioavailability of antibodies and limit systemic toxicity. Functionalized nanosystems conjugated mAb can easily maneuver through dense tumor microenvironments and reach the target, a drawback observed in conventional mAb therapy. PEGylated and non-PEGylated liposomes encapsulating anti-CTLA-4 mAb targeting tested in cancer models have shown efficient localization within 18 h of injection [165]. Low concentrations of adenylate cyclase (AC) inhibitor conjugated with poly(sarcosine)-block-poly(L-glutamic acid γ-benzyl ester) (polypept(o)id) nanoparticle enhances anti-tumor activity in combination with regulatory T-cells. Nanotargeted AC reduces anti-inflammatory myeloid cells and checkpoint receptors on T-cells, preventing tumor immune escape [166]. Thus, nanotechnology serves as a good interface for effective ICI targeting within skin cancers microenvironments (Figure 3).

### 8.4. Nanoparticle-Enabled Adoptive Cell Therapy

The TME targeting of NK cells using adoptive cell transfer (ACT) has gained success due to its orchestrated, specific, and selective function toward various cancers. Many clinical trials have been conducted with effective treatment results and an unmatchable adjuvant to conventional therapies. These clinical trials investigate the potential of adoptive transfer modalities for solid and liquid malignancies (www.clinicaltrials.gov, accessed on 7 January 2023). Adoptive cell transfer of macrophages expressing chimeric antigens has been tested in many tumors [167,168]. Though a promising therapeutic approach, NK-based ACT has encountered several limitations. Attributable to inadequate homing of the cell, low cytotoxicity, limited contact with tumor cells, and the overall immunosuppressive microenvironment. Similarly, macrophage-based adoptive cell therapy has achieved higher success rates in conjunction with nanoparticles. Macrophage-based adoptive therapy conjugated with copper sulfide nanoparticles has demonstrated substantial tumor regression in mouse melanoma models [169]. Thus, nanoparticles enhance ACT irrespective of tumor antigen presentation. Bone marrow-derived macrophages induce reactive oxygen species (ROS) production, increased PD-1 expression, and phagocytic activity when used with intratumoral administration of copper sulfide nanoparticles, subsequently prolonging survival rate in mice models [169]. Further, nanoparticles have facilitated overcoming the limitation associated with T-cell therapy, including restricted intratumoral delivery, suboptimal activation, and intratumoral dysfunction attributed to immunosuppressive TME. Nanoparticles can be functionalized explicitly for robust activation of T-cells ex vivo and conjugated onto T-cells for enhanced T-cell therapy [170] (Figure 3). Liposomal nanoparticles with IL-2 and TGF β can mediate macrophage and NK cell homing and infiltration in the tumor site, and nanocomposite containing IFNγ or IL-2 can facilitate the conversion of immunosuppressive TME to more immunoresponsive environment enhancing NK tumor recognition and killing [171,172]. Recent preclinical advances have benefitted from the using nanoparticles to advance and improve T-cell therapy in various tumors. Magnetic nanoparticles and nanoengagers formulated with phenylboronic acid and IgG and nanoparticle-mediated delivery of CAR and NK-1 have enhanced adoptive cell therapy against solid cancers and melanomas [173].

## 9. Nanoparticle-Aided Personalized Skin Cancer Immunotherapy

Skin cancers can be broadly classified as nonmelanoma and melanoma, each having diverse subtypes. However, at the patient level, every cancer has a unique set of genetic and epigenetic mutations. Furthermore, additional changes are incorporated as cancer proliferates, accumulating different mutations in the different cells within the same cancer microenvironment. Hence, there is a need for effectively tailored therapies suitable for each patient. Though malignant and non-malignant skin cancers have demonstrated sensitivity towards an advanced immunotherapeutic substantiating prospective immunologic cure of skin cancers, many patients develop primary or adaptive resistance. Resistance mechanisms against immunotherapy are generated by a complex interplay of TME, T- cells, cytokines, and existing co-morbidities, which are variable among patients. Hence, personalized immunotherapeutic approaches are considered to hold promise against skin cancers. Adoptive cell therapy, as a customized approach targeting exclusive somatic mutations against melanoma, has resulted in long-lasting regression in patients [174]. With the current tools, genetically engineered T-lymphocytes expressing chimeric antigens can be used as personalized adoptive cell therapy. mRNA-based personalized vaccines that activate T-cell responses specific to a patient’s tumor can amplify the endogenous tumor-specific T-cells’ repertoire. Predicated on neoantigens and TAA-based designs, therapeutic cancer vaccines can be personalized as safe and effective immunogens to drive anticancer immune response [175]. With the effective integration of nanotools in skin cancer immunotherapy, several immunotherapeutic modalities such as vaccines, adoptive cell therapy, immunomodulators, and immune checkpoint inhibitors can be modeled as precision medicines. Nanomaterials facilitate the effective use of personalized cancer immunotherapy by enhancing the immunogenicity of tailored antigens, improving antigen presentation, and stabilizing antigens. Nanoparticles effectively deliver immune agents in stabilized native forms to the target site with high accuracy, facilitating a potent anti-tumor response. Many nanovaccines approved by the FDA are now in clinical trials. A range of advanced immunotherapeutic methodologies can be explored for delivering precision medicine with enhanced outcomes.

## 10. Conclusions and Perspective

Diagnostic and therapeutic modalities for skin cancers have shown promise through immunotherapy. However, immunotherapeutics have also reached their response limit in nonmelanomas and melanomas. This article discusses the current knowledge of the integration of nanoplatforms with the immunotherapeutics regimes that are already in practice and sheds light on how immunotherapy against skin cancers can be enhanced. Coordinated efforts to amalgamate current therapy and diagnosis with nanotechnology to enable robust and sustained responses of skin cancer immunotherapy have proven successful in sporadic studies and clinical trials. Compared to the traditional procedure, immunotherapeutic targeting through nanoparticles or nanoencapsulation allows for better targeting, improved epithelial permeability, specific tumor targeting, enhanced bioavailability, and minimized side effects. Emerging nanosystem-based approaches preclude the importance of integrating nano-immunotherapeutics in skin cancer treatment to accelerate diagnosis and therapy.

## 11. Challenges and Future Directions

Even though nontherapeutic regimes have proven successful in many cancers, incorporating nanoplatforms against skin cancers is nominal. Nanomaterials are expected to greatly improve current skin cancer detection, tumor imaging, and therapy methods while reducing toxicity compared to traditional treatments. Nanomaterial-based immunotherapeutic strategies are expected to enhance the efficacy of immunotherapy as well, obviating the need to explore nano-based precision medicine against skin cancer.

Nevertheless, the inherent modular nature of nanomaterials can result in both beneficial and detrimental effects based on the targeted environment. Recently, studies have reported tissue accumulation, nuclear infiltration, increased oxidative stress, hepato- and nephron-toxicity, tissue-specific inflammation, etc., which are linked to nanomaterial-based therapies. The use of nanomaterials in cancers necessitates the need for nanotoxicology studies before their therapeutic or diagnostic use. Urgent attention to identifying and bridging the gap in the beneficial use of nanomaterials-based therapies and associated toxicities is fundamental to overcoming the current challenges. In sum, numerous nanosystems and strategies have been conceptualized to boost the efficacy of skin cancer immuno-theranostics. Recent years have observed significant advances at the nano–immuno interface in the preclinical settings with encouraging proofs-of-concept attained in the clinical settings.

## Figures and Tables

**Table 1:** The above table lists some types of nanoparticles commonly used in immunotherapeutic modules against various cancers. It lists the modes of surface functionalization (SF) and encapsulation (EN) with drugs, peptides, nucleic acids, etc. The immunotherapeutic and diagnostic functions of each type of nanomaterial are mentioned.

Nanomaterial	Types	Surface Functionalization (SF) and Encapsulation (EN)	Immunotherapeutic and Diagnostic Function
Liposomes	Ethosomes, transfersomes, PEGylated liposomes	SF: Aptamer, antibodies, proteins, peptides, small molecules, carbohydrates EN: Hydrophobic drugs, mRNA, DNA, siRNA, imaging agents	Mechanical damage, receptor mediated targeting, vaccines, checkpoint blockers
Metallic Nanoparticles	Magnetic iron (Fe_3_O_4_), Superparamagnetic iron oxide Nanoparticles (SPION), Gold (AuNP),Silver (AgNP), Glyco-gold NP, Nickle oxid (NiO), Aluminium, Titanium and Zinc oxide Al, TiO, ZnO, Pallidium	SF: Antigens, adjuvants, antibodies, EN: can be encapsulated in liposomes, dendrimers, carbon nanotubes	Photo thermal therapy, Photodynamic visualization TME modulation (ROS generation, hypoxia relief, glutathione depletion, thermal ablation), photothermal editing, hyperthermia, sonodynamic therapy, TME reprogramming, T-cell activation
Carbon Nanotubes	Single walled (SWCNTs), multiwalled (MWCNTs), fullerene encapsulated carbon nanotubes	SF: shRNA, aptamers, mAb, immunotargeting short peptides, growth factors, intracellular targeting tags EN: Fullerenes	Immunoactive compounds (genes and proteins), tumor imaging, photothermal therapy, drug and vaccine delivery, nanovaccines, antioxidants subcellular targeting, biosensing
Polymeric Nanoparticles	Poly(lactide) (PLA) Poly(lactide-co-glycolide) (PLGA) copolymers, Poly (ɛ-caprolactone) (PCL) Poly(amino acids) –poly-L-lysine, poly-L-arginine	SF: Subcellular targeting chimeric proteins, pH sensitive hybrid membrane, antibodies EN: Drugs, antigenic proteins, tumor associated antigens, aptamers and cellular receptors	Cancer vaccines, immune checkpoint inhibitors, drug targeting, TME targeting, biosensing, bioimaging, antigen presentation, antigen internalization, TME modulation, fluorescence and photoacoustic imaging
Dendrimers	Polyamidoamine (PAMAM) dendrimers Poly(propylene imine) (PPI) dendrimers Citric acid dendrimers Polyester dendrimer system Polyether dendrimers. Phosphorous dendrimers Glycodendrimers Carbosilane dendrimers.	SF: Imaging probes, drug, ligand, nucleic acid, cell anchors, controlled release substances, gene delivery; stimuli-response; targeted drug delivery EN: drugs, genetic material, mRNA	Vaccines, antibodies and immunostimulation, imaging, adjuvant, antigen presentation, antigen internalization, TME manipulation, adoptive cell transfer, immunomodulation, checkpoint inhibitors

## Data Availability

Not applicable.
